# Unlocking Instructors’ Assessment Insights: General Chemistry Instructors’ Perspectives on Types of Questions and their Classroom Application

**DOI:** 10.1021/acs.jchemed.5c00116

**Published:** 2025-09-03

**Authors:** Emily A. Kable, Ying Wang, Lu Shi, Marilyne Stains

**Affiliations:** † Department of Chemistry, 2358 University of Virginia, Charlottesville, Virginia 22904, United States; ‡ STEM Education Innovation and Research Institute, 10668 Indiana University Indianapolis, Indianapolis, Indiana 27412, United States

**Keywords:** Assessment, Instructor Thinking, Assessment Design, Chemistry Education Research, General Chemistry

## Abstract

Assessment communicates to students the takeaways from a course. Unfortunately, studies have demonstrated that assessment in general chemistry courses typically includes lower-cognitive demand questioning, such as recalling information and calculation-based questions. To support chemistry instructors’ inclusion of higher-cognitive demand questions, chemistry education researchers have developed research-based assessment tools (e.g., Three-Dimensional Learning -3DL - and concept inventory). However, previous reports have highlighted a low uptake of these tools. To explore the reasons behind this slow adoption, instructors’ thinking about these types of assessment tools should be probed. In this study, semi-structured interviews were conducted with 19 general chemistry instructors to explore whether and how instructors would use four different types of multiple-choice questions, including a standard conceptual question, a calculation-based question, a 3DL question, and a concept inventory-type question in their courses’ exam/midterm, homework, and/or in-class activity. Instructors in this study were interested in using the research-based assessment tools in at least one assessment context (i.e., home, in-class, or exam). The most common modification described by the instructors across the four types of questions was shifting the format from close to open-ended as it allows instructors to better understand student thinking and can promote better conversations among students in class settings. Finally, the analysis of interviews shows variations in instructors’ expectations for the cognitive demand of questions on exams. Taken together, these findings suggest a need to further probe instructors’ assessment literacy to inform the development of professional development programs and policies that would support higher-quality assessment in general chemistry courses.

## Graphical abstract

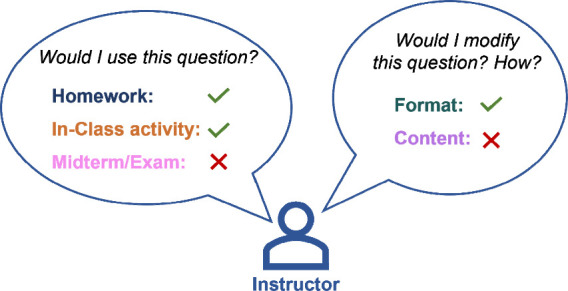

## Introduction

One of the major goals of discipline-based education research has been to develop student scientific literacy.^1^ Scientific literacy is defined as an individual’s ability to read, understand, and communicate opinions on scientific topics.^2^ Extensive research efforts have been focused on developing innovative instructional strategies^3,4^ and curricula^5–8^ to support students’ conceptual understanding^9,10^ and scientific practices, core features of scientific literacy.^11,12^ While these efforts have led to some positive student outcomes,^13–21^ researchers have argued that solely focusing on instructional strategies and curriculum is not enough, and that there needs to be an equal focus on how students are being assessed.^22,23^ As Shepard^24^ indicated in their work, assessment practices should be reformed to align with instructional practices to support student learning.^24^ Indeed, content assessed in a course communicates to students what instructors deem important for them to learn.^12,25,26^ A misalignment between the curriculum implemented, the educational purpose of innovative instructional strategies, and the assessment employed can undermine the impact of instructional innovations on student learning.

The focus of assessment reforms in chemistry education has mostly been on the development of research-based assessment tools. These assessment tools are sets of questions measuring cognitive and noncognitive factors that have been empirically developed and are intended to inform classroom practice.^27^ For example, numerous tools measuring students’ conceptual understanding of particular chemistry concepts, i.e. concept inventories, have been developed by chemical education researchers.^28^ Moreover, instructors can now leverage a framework that supports them in designing questions that assess scientific practices alongside core concepts.^29^ However, the uptake of these tools has been limited. For example, a national survey of chemistry instructors on their assessment practices shows that about 28% of the 829 survey respondents used concept inventories.^27^ Studies have also reported on chemistry instructors’ reliance on questions with lower cognitive demand (e.g., recall, direct application of rule).^12,25,30–32^ A potential reason for the lack of reform in the assessment practices of chemistry instructors might be our limited understanding of their thinking about and knowledge of assessment. This study aims to address this dearth of knowledge by exploring general chemistry instructors’ perceptions of different types of questions and their potential integration in the assessment plan for their course.

### Assessment in General Chemistry Do Not Typically Focus on Higher-Level Thinking

Several studies have explored the nature of assessment questions (e.g., exams and homework questions) in general chemistry courses based on their cognitive demand or the extent to which they assess students’ conceptual understanding.^12,22,31,32^ For example, Dávila and Talanquer^32^ characterized the end-of-chapter questions in three top-selling general chemistry textbooks and a first-semester general chemistry American Chemical Society (ACS) exam using Bloom’s Taxonomy.^33,34^ They found that the majority of questions were classified at the intermediate level of Bloom’s Taxonomy (i.e., application, analysis) and lacked diversity in their cognitive engagement (e.g., application questions were mostly algorithmic). An algorithmic question typically involves solving a problem via mathematical calculations.^10^ Only a small proportion of questions were classified in higher cognitive levels (e.g., requiring students to apply their knowledge in new contexts).^32^ Shah et al.^31^ characterized assessment questions in a second-semester general chemistry course over four different semesters and found that calculation-based questions were the most prevalent style of question.^31^ Therefore, assessment questions in general chemistry courses do not seem to emphasize high-order cognitive skills, the kind that would promote conceptual understanding. Instead, they overemphasize algorithmic thinking. These types of questions can marginalize students’ success in general chemistry as their ability to answer these questions is tied to their access to precollege mathematics programs.^31,35^ For example, Shah et al.^31^ compared the performance between students who were considered to be at-risk based on their math placement exams and students who were not considered at-risk. They found that calculation-based and basic-recall questions contributed the most to the difference in overall assessment scores between these two groups of students.^31^ Additionally, the results from this study showed that the students classified as at-risk performed similarly to the not-at-risk students on higher cognitive level questions (e.g., inferring or predicting questions).^31^ Therefore, students in general chemistry courses have limited opportunities to demonstrate their conceptual understanding of chemistry concepts and are sent strong signals to memorize rules and facts as well as work on their mathematical skills instead.

### Novel Assessment Tools Aimed at Promoting Conceptual Understanding and/or Scientific Practices Have Been Developed

Chemistry education researchers have developed assessment tools that aim to support instructors in their ability to assess students’ conceptual understanding (i.e., students’ ability to reason about chemical concepts beyond rote learning, problem-solve, predict or explain chemical phenomena, translate their understanding using chemical representations, and applying their understanding to new contexts^9^) and scientific practices.^22,28,36^ Two examples of these tools are concept inventories and the Three-Dimensional Learning Assessment Protocol (3D-LAP).

#### Concept Inventories

Concept inventories consist of multiple-choice questions that aim to assess student understanding of concepts. Questions and distractors have typically been developed from extensive student interviews that aim to identify their alternative conceptions about specific chemical concepts.^28^ Numerous concept inventories have been developed in chemistry^37,38^ since the first published one in 2002.^39^ These inventories often include questions that probe students’ understanding of concepts via visualizations, such as particulate representations. As was mentioned earlier, a national survey has demonstrated that the uptake of these inventories by chemistry instructors has been slow.^27^ Interestingly, this study found that instructors who were more likely to use research-based assessment tools such as concept inventories were also those who had previously attended a teaching-focused workshop.^27^

#### Three-Dimensional Learning Assessment Questions

More recently, chemistry education researchers have developed assessments that align with the Three-Dimensional Learning (3DL) framework that was originally presented in the Next Generation Science Standards (NGSS) in 2012.^11^ The core of the 3DL framework is to incorporate three main dimensions in a question: core scientific ideas, scientific and engineering practices, and crosscutting concepts. Core scientific ideas center on electrostatic/bonding interactions, atomic/molecular structures and properties, energy, and change and stability in chemical systems.^11,29^ Scientific and engineering practices incorporate real-world practices for investigating problems, such as analyzing and interpreting data or constructing explanations.^11,29^ Crosscutting concepts add an interdisciplinary approach by requiring students to think about different aspects of a topic such as structure and function.^11,29^ When combined, these three dimensions encourage students to think more conceptually about various scientific phenomena. To help instructors develop and assess whether their current questions align with the framework, a Three-Dimensional Learning Assessment Protocol (3DLAP) has been developed.^22,29^ Few studies have explored the preponderance of 3DL questions in different assessment contexts (e.g., in-class activity, homework, or midterm exam) in chemistry courses. Stowe et al.^12^ evaluated the ability of questions on midterm exams in three different general chemistry courses to elicit 3DL using the 3DLAP.^12^ They found that the majority of the assessment questions did not elicit 3DL. A similar study conducted in organic chemistry found similar results.^40^ Work has been done to explore factors associated with instructors’ adoption of 3DL questions.^41^ The study found that biology instructors who rely on Bloom’s Taxonomy to write their questions were more likely to write 3DL questions. Nelson et al.^42^ characterized STEM instructors’ motivation to adopt 3DL questions within their courses. One of the major findings from this study was that faculty motivation to implement 3DL questions was due to the ability of 3DL questions to support student learning. In both studies, participants highlighted the difficulty and time commitment required to write 3DL questions, which may constitute a barrier to adoption.

### Instructors’ Assessment Literacy

To understand the reasons for instructors’ lack of adoption of research-based assessment tools, it is important to understand instructors’ thinking about assessment within their course.^43^ Buckley^43^ argues that understanding instructors’ thinking on assessment can help bridge the gap between research and practice.^43^ An important component of instructors’ thinking on assessment is their assessment literacy, which is defined as the skills and knowledge that an instructor needs to effectively utilize assessment tools within their course.^44^ Abell and Siegel^45^ developed a model based on prior literature^46,47^ and empirical studies^48,49^ to conceptualize assessment literacy. This model consists of four types of knowledge: knowledge of assessment purposes, knowledge of what to assess, knowledge of assessment strategies, and knowledge of assessment interpretation and action-taking.^45^

Instructors’ *purpose for assessing* has been categorized in three main categories: assessment of learning (AoL), assessment as learning (AaL), and assessment for learning (AfL).^50^ Instructors who believe a purpose for assessment is AoL use assessments to ensure students have achieved certain learning outcomes (i.e., summative assessments like midterms and final exams).^50,51^ Instructors who believe the purpose for assessment is AaL see assessments as a means to promote students metacognitive skills related to their learning process (e.g., muddiest point).^50,51^ Lastly, instructors who believe the purpose for assessment is AfL use assessments to provide feedback to both students and instructors and leverage this feedback to modify instruction wherever deemed necessary (e.g., clicker questions).^50,51^ Recent work has captured 19 general chemistry instructors’ assessment practices and their conceptions of purposes for assessing.^52^ This study indicated that all but one instructor viewed assessment as an evaluation of learning (AoL), a third of their sample viewed assessment as a method to promote metacognitive learning skills (AaL), and a third of their sample viewed assessment as a tool to provide feedback to students and instructors (AfL).^52^ Additionally, this work revealed that very few general chemistry instructors described all three of these purposes for assessment. However, instructors who described more than one purpose for assessment implemented a greater variety of assessment contexts.^52^

An instructor’s *knowledge of what to assess* entails what instructors should assess in relation to their curricular goals and what they view as important to learn in their course.^45^ For example, if instructors believe that assessment should focus more on students’ conceptual understanding and scientific practices, then their assessment should reflect that belief. Sanabria-Ríos and Bretz^30^ investigated 10 general chemistry professors to understand the extent to which their exams aligned with their learning objectives. The results highlighted that instructors wrote exam questions that aligned with their course objectives, and that the instructors cognitive expectations (i.e., attitudes and beliefs about learning chemistry) aligned with the conceptual and algorithmic nature of their assessments.^30^

An instructor’s *knowledge of assessment strategies* relates to how instructors assess their students’ learning progression through a unit.^45^ This includes instructors needing to know what different assessment strategies they can utilize in their course, and how they design these strategies.^45^ There is limited research within chemistry on instructors’ knowledge of assessment strategies. When considering the different assessment strategies that instructors can use, it is also important to think of how they design their different assessment tools. Assessment design is the process that takes place when instructors organize specific assessment tools for a course including the selection of assessment questions to be used within an assessment tool, timing of the assessment tools, the development of rubrics or grading scheme, and any modifications made to their assessment questions based on student outcomes.^53^ Within chemistry, assessment design remains relatively unexplored; however, it has been previously reported that chemistry instructors have autonomy in their assessment design process.^54^ Overall, chemistry instructors’ *knowledge of assessment strategies* has been relatively unexplored.

Finally, instructors also need to know how to make sense of the results from the different assessment tools and the actions they should take in response to these results.^45^ For example, this can include instructors making modifications to their instruction to help support student learning based on how students performed. Within secondary education, high school chemistry teachers have shown to use student performance on different assessment tools to interpret student understanding but did not communicate an action in relation to their instruction in response.^55^ This fourth dimension of assessment literacy is also underexplored in chemistry education.

## Research Questions

A better understanding of chemistry instructors’ assessment literacy can help in developing professional development programs and resources that would enhance their literacy and thus impact their assessment practices.^56^ In this study, we aim to understand chemistry instructors’ perceptions of different types of assessment questions, including a standard conceptual, a calculation-based, a 3DL, and a concept inventory-type question, as a means to get insight into training strategies and resources that could enhance the adoption of assessment tools promoting scientific literacy. As such, the following two research questions guided this study:

1. How do general chemistry instructors perceive the potential usage of different types of assessment questions in homework, in-class activity, and/or midterm/exams in their course?
2. How do general chemistry instructors describe modifying assessment questions before implementing them in their course?

## Methods

This study, conducted in the Spring of 2021, was approved by the institutional review board at the University of Virginia: Study 001406.

### Participant Recruitment

A convenience sample of 143 general chemistry instructors were recruited via email. Emails were collected on chemistry departmental Web sites. These instructors spanned 51 institutions across two states that are geographically close to the University of Virginia. In total, 19 instructors from 14 different institutions (Table 1) agreed to participate in the study. The instructors taught either small (classified as less than 50 students) or large (classified as more than 100 students) general chemistry courses that ranged from approximately 15–500 students (Table 1). The demographics of the participants including gender, academic rank, and teaching experience are shown in Table 2.

**Table 1:** Participants’ Context

Institution type^a^	Number of participants	Class size	Number of participants
R1	6	Less than 50	12
Masters	8	More than 100	7
BAC	5		

a Institution type is based on the Carnegie Classifications:^57^ R1: Doctoral Universities- Very High Research Activity. Masters: Master’s Colleges and Universities. BAC: Baccalaureate Colleges- Arts and Sciences Focus.

**Table 2:** Participants’ Demographics

Demographic variables		Number of participants
Gender	Female	11
	Male	8
Academic Rank	Lecturer	7
	Associate Professor	6
	Professor	4
	Assistant Professor	1
	Visiting Professor	1
Teaching Experience	0–5 years	5
	6–10 years	5
	11–15 years	4
	16 or more years	5

### Data Collection

The current study is based on a portion of a larger interview study.^52^ The semi-structured interview protocol was designed by LS and MS and interviews were conducted by LS via Zoom. The interviews ranged between 47 to 93 min. The relevant portion of the interview protocol that informs this study is in the .

The assessment questions developed and selected for this study focus on the concept of chemical equilibrium as it has been identified as an important topic within General Chemistry by different institutes, such as the ACS^57,58^ and the NGSS Framework.^11^ This importance is reflected in the ubiquity of coverage of this topic in general chemistry textbooks. We thus thought that this would be a common topic taught and assessed by all the study participants, which allowed us to control the topic being assessed as a factor for this study.

This study explores instructors’ perspectives regarding four multiple-choice questions. Multiple-choice format was chosen due to its frequent use in large enrollment courses.^59,60^ We also wanted to control for question format to provide opportunities for instructors to focus on similarities and differences in the content (e.g., concept and skill) that was assessed in the questions rather than differences in format. The four multiple-choice questions focused on Le Chatelier’s principle and all but one use the same reaction - the dissociation of dinitrogen tetroxide into two nitrogen dioxide molecules. Three questions (a standard conceptual, calculation-based, and concept- inventory type question) that were probed during the interview were adapted from a McGraw Hill textbook.^61^ McGraw Hill did not permit reproduction of these questions and therefore we provide descriptions instead. The standard conceptual question asks students to predict how the equilibrium would shift when nitrogen dioxide is added to the reaction. The calculation-based question asks students to calculate the mass of nitrogen dioxide produced after more of it had been added to the equilibrium reaction. The third question from the textbook was a particulate-style question similar to those that can be found in some concept inventories. This question provides a reaction mixture consisting of white and black spheres and the chemical reaction (white spheres become black spheres in a one-to-one ratio). The question asks students to identify which of the five pictorial representations of the reaction mixture depicts the reaction at equilibrium after more black spheres have been added. The fourth question was a 3DL question (Figure 1), which was adapted from an existing 3DL question in order to have consistency in the reaction being used for each question.^29^ This question assesses students’ understanding of a core idea - *Change and Stability in Chemical Systems*, a crosscutting concept - *Cause and Effect: Mechanism and Explanation, and* a scientific and engineering practice - *Construct Explanations*.^29^

**Figure 1:**
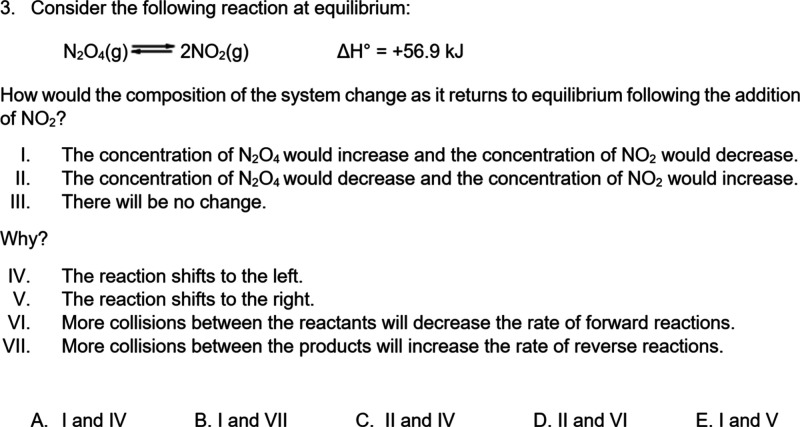
Three-dimensional learning (3DL) question adapted from Underwood et al. (2017).^29^ Copyright [2017] American Chemical Society.

During the interview, instructors were provided with all four questions, without any description of the assessment questions, and a Venn Diagram (Figure 2). They were asked whether they would include each of the questions in different assessment contexts (i.e., their course’s homework assignment, in-class activity, and/or exam/midterm). If instructors could not envision using a particular question in their course, they were told to place it outside the Venn diagram. Furthermore, instructors were asked whether and why they would make any modifications and what the nature of these modifications would be. Each interview was audio-recorded and transcribed verbatim using Temi with all identifiable information excluded for each study participant prior to analysis.

**Figure 2:**
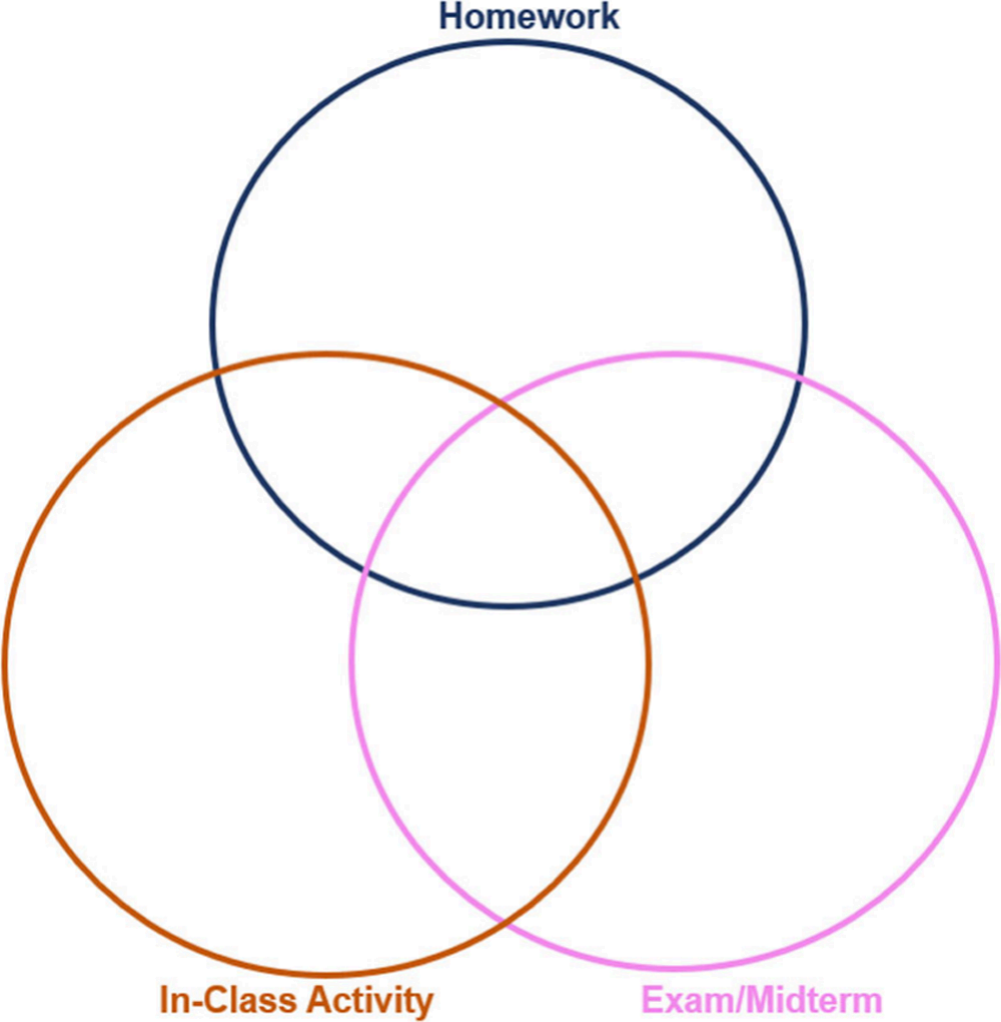
Venn diagram provided to the participants to help them indicate in which assessment context they would use the questions provided.

### Data Analysis

Interview transcripts were analyzed via thematic analysis. Thematic analysis is a qualitative research method that entails analyzing data to look for patterns or themes.^62^ To identify patterns or themes, the transcripts were first memoed by EAK and YW; this allowed for data familiarization and the preliminary identification of patterns and themes. Those memos were then used to do an inductive coding process, which entails looking at the interview transcript data and developing codes.^62,63^ The codes collectively formed an initial codebook. The initial codebook was then iteratively refined by two coders (EAK and YW) using 1–2 transcripts at a time, until minimal modifications were made to the codebook. After each codebook iteration, EAK and YW met to discuss disagreements between codes, clarify definitions and modify codes. Summaries of each iteration of the codebook refinement are detailed in the . The final codebook is also provided there.

In this study, intercoder reliability (ICR) was conducted by EAK and YW. ICR was established by independently coding with the final codebook ∼20% of the transcripts (4 out of 19 instructors), two that had previously been used to modify the codebook and two new transcripts. One metric of ICR is percent agreement which is the proportion of agreement between coders after independently coding transcripts.^64^ The percent agreement was 85%, which exceeds the accepted standard of 80%.^65^ A limitation to solely using a percent agreement to establish ICR is that percent agreement does not take into account the random chance of agreement; therefore, Cohen’s kappa was calculated to account for chance agreement.^64^ The Cohen’s kappa for the four transcripts was 0.84, which indicates strong agreement between the two coders.^64,66^ With evidence of reliability, the remaining ∼80% (15 instructors) of the transcripts were coded independently by EAK. Any confusions that came up while coding the transcripts were discussed with YW and MS. After independently coding the remaining transcripts, the research team (EAK, YW, and MS) met to see what codes could be grouped into larger overarching categories. To code the rationales for instructors’ modifications to each of the questions, a consensus agreement was reached between EAK and YW.

In the Results section, we discuss rationales for integration of a question in instructors’ assessment plans that were discussed by at least four instructors (>20% of the sample); we discuss rationales for lack of integration of question in assessment plans that were discussed by at least two instructors (>10% of the sample) due to less instructors falling into this category. Lastly, we report on the most common rationales that supported instructors’ interest in modifying a question. Full analysis of instructors’ rationales for selecting or not selecting a question, and modifications are available in .

### Trustworthiness

Within this study, we followed Lincoln and Guba’s recommendations to establish the trustworthiness of the data analysis process.^67^

#### Credibility

Credibility is the confidence that the findings are true to the participant’s view.^67–69^ Credibility was established by having multiple investigators analyze the data. Two researchers (LS and MS) developed the study and interview protocol, and two researchers (EAK and YW) contributed to the development and refinement of the codebook to provide multiple perspectives on the data analysis. Lastly, peer debriefs were conducted with researchers not involved in the data analysis process to talk through interpretations of the data analysis process and data interpretations from the frequency of codes.

#### Transferability

Transferability of data allows for the potential consideration of the conclusions of the current study to be transferred into a different setting.^67–69^ Transferability was established within this study by providing a rich description of the methodology and data analysis process as detailed above.

#### Dependability

Dependability is the consistency in which the findings from the data are within a study over time.^67–69^ Dependability was established through stepwise replication done through the intercoder reliability process. For a detailed explanation of this process, see the data analysis section above.

#### Confirmability

Confirmability is the extent to which the data can be interpreted by different researchers in similar ways.^67–69^ Confirmability was established through having two researchers iteratively and independently coding the interview transcripts to finalize the codebook. Additionally, having an audit trail of each version of the codebook helps establish confirmability.

## Results

The goal of this study was to characterize general chemistry instructors’ preferences regarding different types of multiple-choice questions being utilized in their course as either homework, in-class activity, and/or exam/midterm. Additionally, this study explored whether, how and why instructors would want to make modifications to these questions.

### Standard Conceptual Question

#### Fit with Assessment Context

Fourteen of the 19 instructors placed this question in the exam/midterm portion of the Venn diagram, 12 in the homework portion and nine in the in-class activity portion. Few of the instructors (*n* = 4) stated that they would use this question in all three assessment contexts, and five instructors suggested that they would only use this question in one type of assessment – exam mostly (Figure 3).

**Figure 3:**
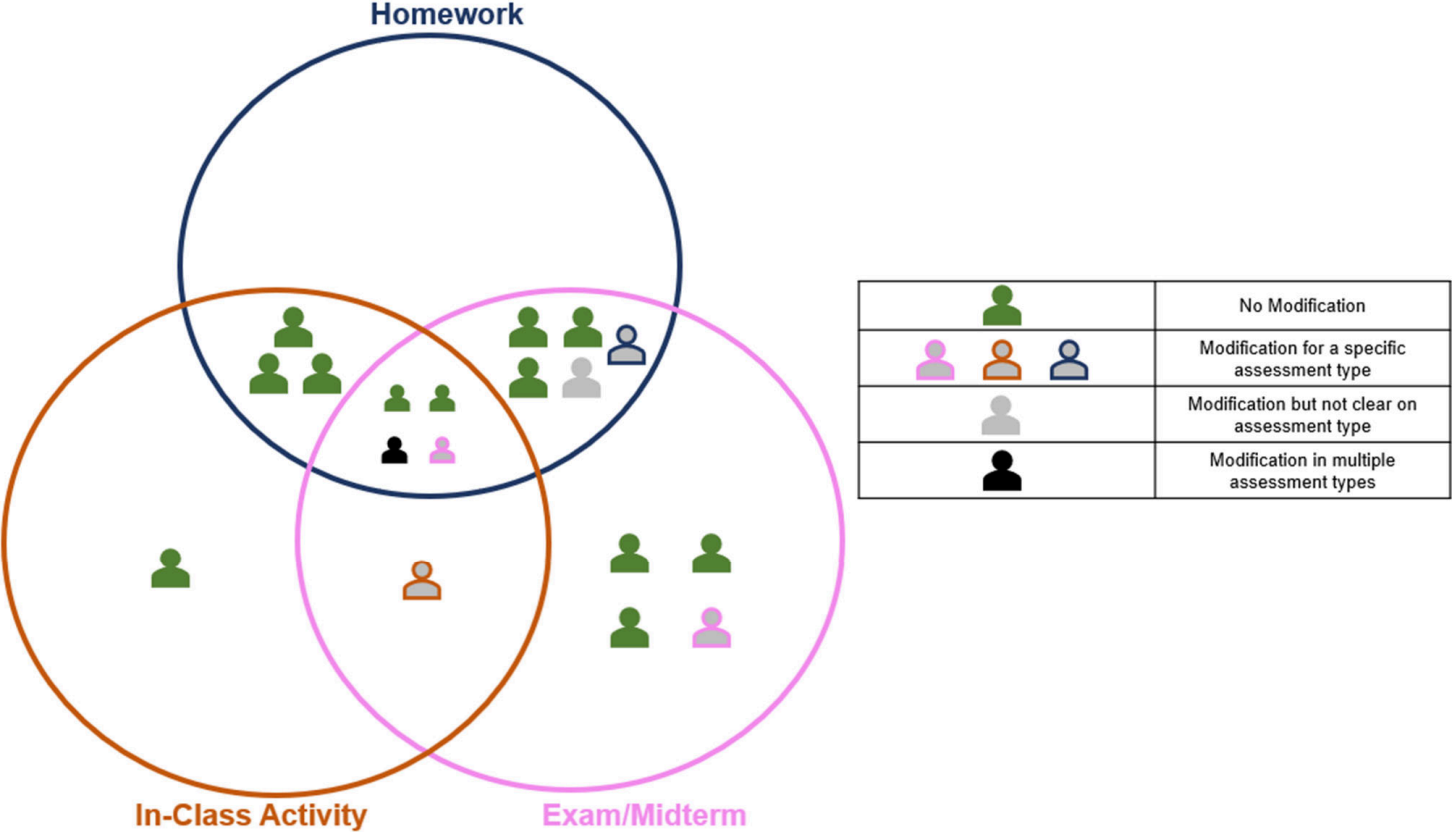
Instructors’ use for the standard conceptual question.

Eleven instructors indicated an interest in using this question across the different assessment contexts because they felt it was ‘easy to answer’ for the students, as exemplified by Instructor 18:

*“So, this [standard conceptual question] is definitely the type of question that I would ask. I could see myself asking it in all of these situations. It’s not a particularly hard question, which makes it ... good as kind of an entry level for an in-class activity. Um, and homework is a lot of practice. So ... you need some questions at this level. And I also like questions like this on the exam or the midterm.” – Instructor 18*

Another common rationale was that the question allows for ‘easy grading’ (*n* = 5), as shown by Instructor 13:

*“It [standard conceptual question] lends itself to in class and homework, because it’s a fairly low-level kind of question, and it is multiple choice so, it’s easily gradable.” – Instructor 13*

Some instructors (*n* = 6) explicitly indicated that they would not use this question for a particular assessment context (Figure 4). Four instructors described not wanting to use this question for an in-class activity, two did not want to use it for an exam/midterm; however, no instructor indicated not wanting to use this question on a homework assignment. Interestingly, three instructors explicitly mentioned not wanting to use this question because they felt it was ‘easy to answer’ as Instructor 4 explains:

*“I mean [the standard conceptual question] is similar to you know some of these other cases, but it’s a little bit simpler, I guess. . . I don’t think it’s complex enough for it to be useful for in-class activity ... there’s not enough discussion there. ... It’s more of a ... test of knowledge, without as much of the explanation.” – Instructor 4*

**Figure 4:**
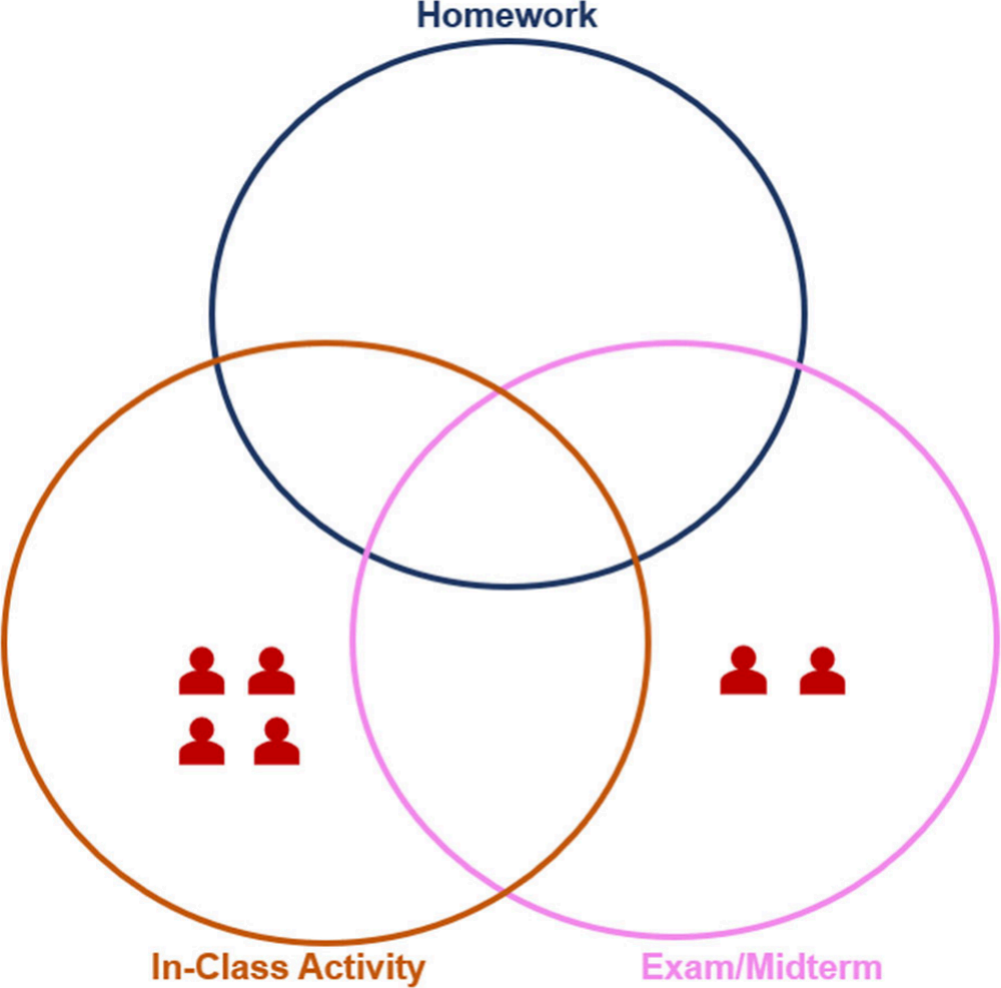
Type of assessment for which instructors would not use the standard conceptual question.

#### Modifications

Only six instructors indicated that they would modify the question prior to utilization in their course (Figure 3). The modifications they described fell into two categories: modifying format (e.g., modifying the question type) and/or modifying content (e.g., modifying the language used in the question). Instructors indicated wanting to modify the format as well as the content of the question (Figure 5). Instructors wanted to modify the format of the question by either making it open-ended or by altering the number of answer choices.

**Figure 5:**
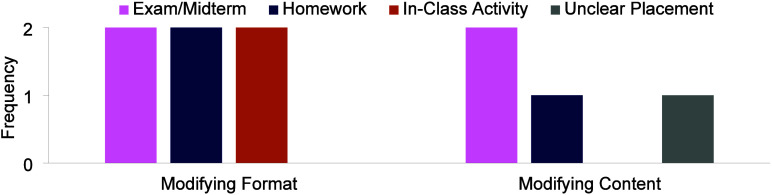
Instructors’ modifications of the standard conceptual question.

*“For instance, if I had a question in class, the first question [standard conceptual question] wouldn’t be multiple choice. ... I would just say okay, ... if you add NO2 what will happen to equilibrium, and they write about it, instead of choosing.” – Instructor 3*

With respect to the content of the question, instructors discussed wanting to modify for an exam or homework either the language in the question or the answer choices. Only two of the six instructors who indicated wanting to modify the question provided a rationale. Both described wanting to prevent guessing or easy elimination by students, as instructor 10 indicates:

*“I feel like [answer choice] A and B are almost like the same response; it’s just the language is a little different, and ... so I feel like a lot of students would immediately like alright it can’t be either one of those two.” – Instructor 10*

### Calculation-Based Question

#### Fit with Assessment Context

Contrary to the standard conceptual question, the majority of the instructors (*n* = 12) would use the calculation-based question in all three assessment contexts (Figure 6). Fifteen of the 19 instructors placed this question in the exam/midterm portion of the Venn diagram, 16 in the homework portion, and 15 in the in-class activity portion.

**Figure 6:**
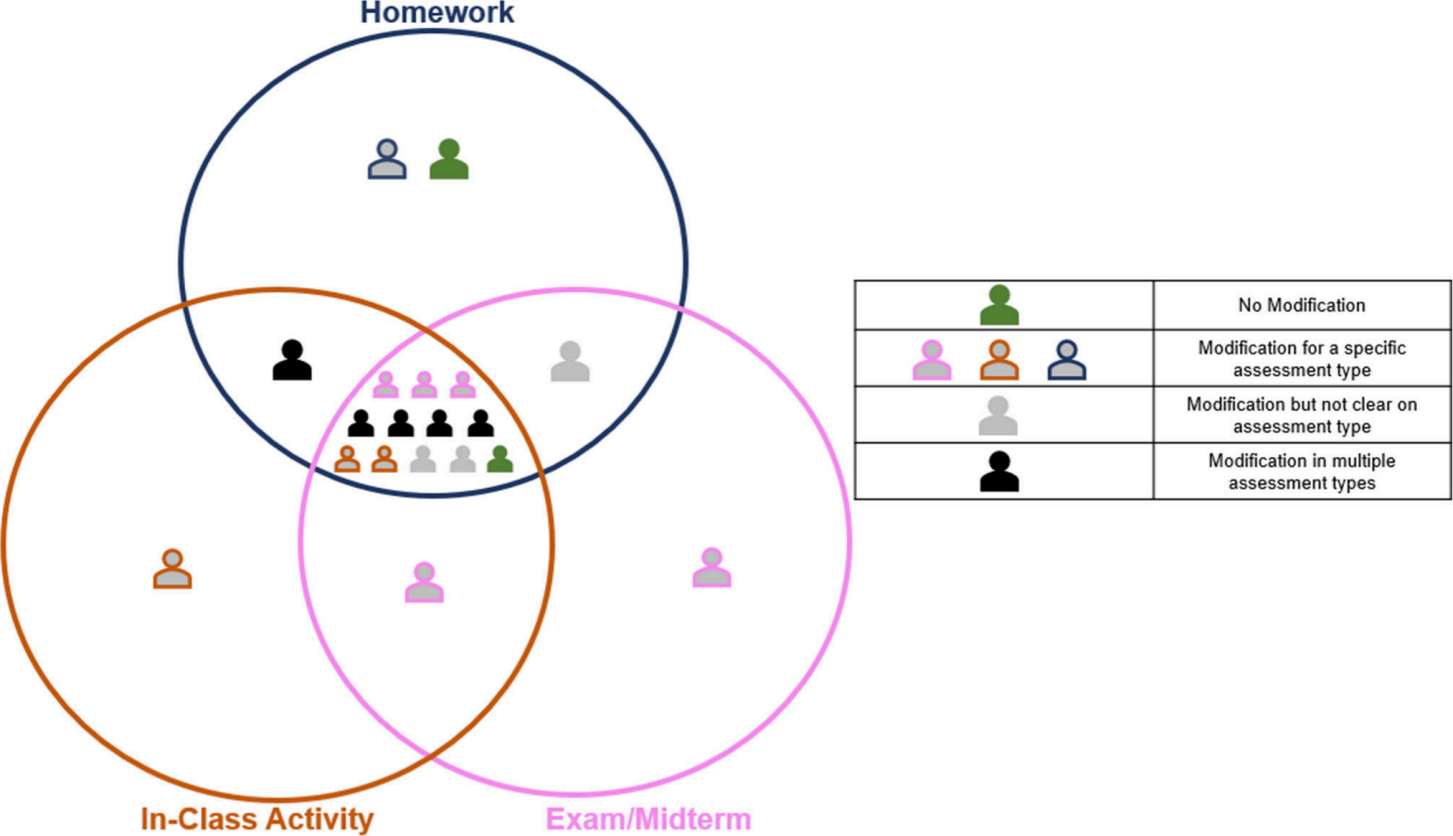
Instructor’s use for the standard calculation-based question.

When discussing rationales for inclusion of the calculation-based question in their courses, instructors mainly focused on in-class activity and homework. Six instructors believe this question provides opportunities for students to discuss their ideas either as part of an in-class activity or via homework by discussing with their peers or asking a TA or instructor for help with the question:

*“I feel like this is the type of question that would be an okay homework question for them to wrestle with, especially if they could ask a TA or an instructor for help with it.” – Instructor 6*

Additionally, five instructors believe this question provides opportunities for ‘student thinking’ for an in-class activity, midterm/exam, and/or homework, which includes students thinking through the question, performing a calculation, or students understanding why they got the answer correct or incorrect, as Instructor 1 elaborates:

*“[Calculation-based question] would be something I would put up and have them do group work. Once a calculator is involved, I put them in groups together and I make some work it out. I give them like you know seven minutes and then they have to agree in a group and answer, and then we talked about the two most common answers. And like so if you answered this is what you did wrong.” – Instructor 1*

Four instructors also discussed wanting to use this calculation-based question in their course for a homework or in-class activity because it is a ‘longer question’ as exemplified by Instructor 3:

*“[Calculation-based question] is a, I would say it’s a learning teams [in-class group work] question, a homework question because it takes a little longer for them.” – Instructor 3*

Four instructors stated they would not use this question for an exam/midterm or an in-class activity (Figure 7). The major reason for this exclusion was that they believe the question requires too much time to complete for these time-constrained assessment contexts as Instructor 15 articulates:

**Figure 7:**
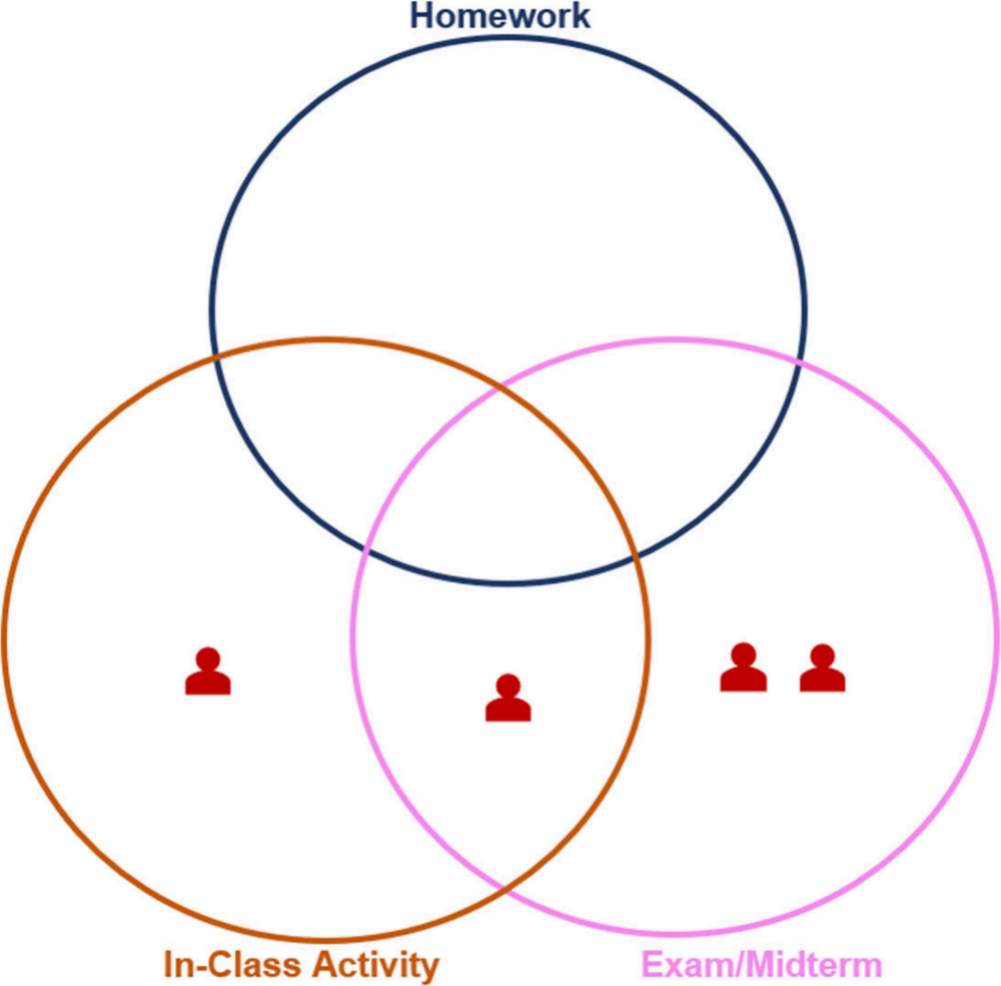
Type of assessment for which instructors would not use the standard calculation-based question.

*“[Calculation-based question] maybe as a homework problem ... There’s a lot of calculations and slows them down on a test. I would be reluctant to do that on a test. It’s a good homework prompt that it’s fairly complicated. I suppose they would do that with an ICE table sort of thing. I would not do it in class for the same reason. I wouldn’t give it on tests. It takes too long.” – Instructor 15*

#### Modifications

Almost all instructors (*n* = 17) indicated a desire to modify the calculation-based question prior to implementing it in their course (Figure 6). The modifications they described fell into three categories: modifying format (e.g., modifying the question type), modifying content (e.g., modifying the language used in the question), or unclear modifications (e.g., instructors mentioned wanting to modify the question, but the details of the modifications were unclear). The most common modification that instructors wanted to make was to the format of the question (Figure 8). Eleven instructors discussed wanting to make the question open-ended as described by Instructor 14:

**Figure 8:**
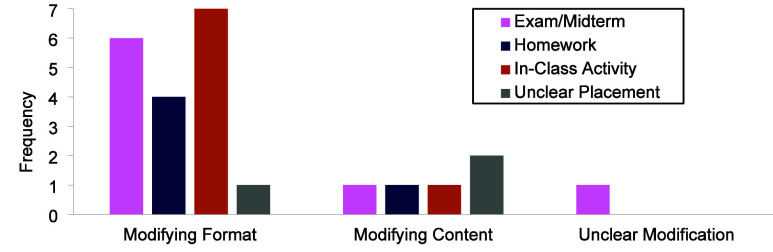
Instructors’ modifications for the standard calculation-based question.

*“Although I wouldn’t normally give [Calculation-based question] as a multiple choice, as a free answer.” – Instructor 14*

The most common rationale for this modification (*n* = 6) was that instructors wanted to see their students’ thinking or work:

*“I would probably want to see the math that my students are doing to solve that problem which they might not do if it’s a multiple choice.” – Instructor 7*

### Three-Dimensional Learning Question

#### Fit with Assessment Context

Thirteen of the 19 instructors placed the 3DL question in the exam/midterm portion of the Venn diagram, eight in the homework portion, and 12 in the in-class activity portion. Four instructors indicated wanting to use this question in all three assessment contexts, and almost half (*n* = 9) would use the 3DL question in only one assessment context, with exam/midterm being the most common (Figure 9). Instructors who would use the 3DL question in their course were more likely to use it for an in-class activity as well as for an exam/midterm than as a homework question (Figure 9).

**Figure 9:**
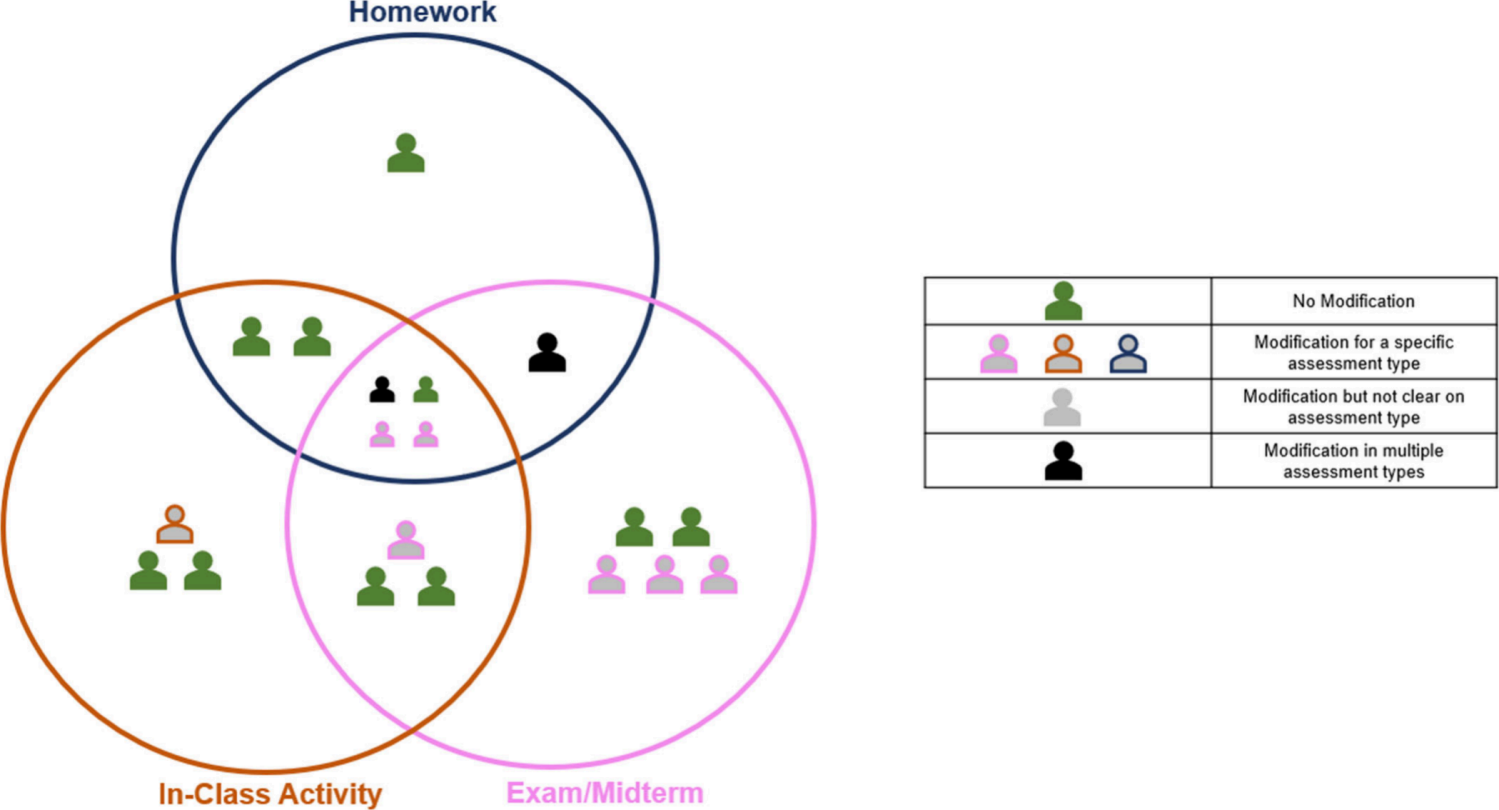
Instructor’s use for three-dimensional learning question.

Several instructors (*n* = 11) mentioned wanting to use this question because it was perceived to be cognitively challenging since students have to explain their answer, and/or because of the built-in, two-part structure of the question as Instructor 2 expresses:

*“I like how the [3DL] is the more challenging question; I mean they have to be able to pick one, two, or three and also tie in an explanation; so I think it’s a deeper level, harder question.” – Instructor 2*

Another rationale that six instructors provided was that it would be a good discussion question for an in-class activity as Instructor 17 discusses:

*“I would put this in an in-class activity because I think this would generate a lot of good conversation in class.” – Instructor 17*

Compared to the other three assessment questions, more instructors (*n* = 9) explicitly stated not wanting to use this 3DL question for a particular assessment context. Six instructors described not wanting to use this question for an exam/midterm, four instructors would not use this question in a homework assignment, and three instructors would not use this question for an in-class activity (Figure 10). Interestingly, six instructors stated they would not use this assessment question for a similar reason as those expressing interest in using the question, i.e. this is a cognitively challenging question:

**Figure 10:**
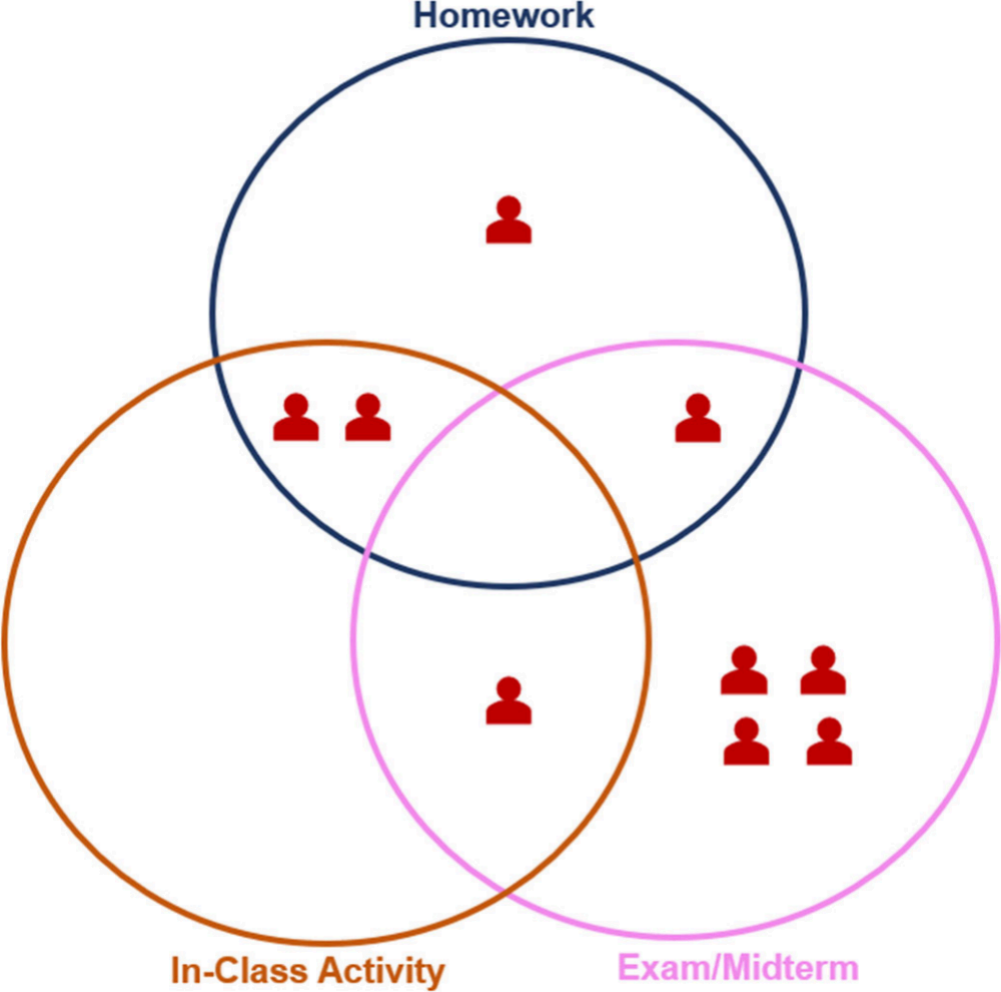
Type of assessment for which instructors would not use the three-dimensional learning question.

*“I don’t know that I would put it on my exam because I guess that those exams are a bit lower level.” – Instructor 18*

#### Modifications

Nine instructors discussed wanting to modify the 3DL question before implementing it in their course (Figure 9). The modifications they described fell into three categories: modifying format (e.g., modifying the question type), modifying content (e.g., modifying the language used in the question), or unclear modifications (e.g., instructors mentioned wanting to modify the question, but the details of the modifications were unclear). The majority of instructors who wanted to modify the 3DL question would do so for an exam/midterm assessment (Figure 11). They indicated wanting to change the format of the question to be an open-ended question or break up the question.

**Figure 11:**
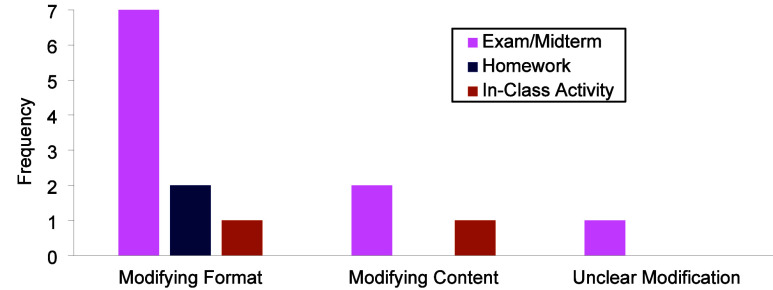
Instructors’ modifications for the three-dimensional learning question.

*“For [3DL question], I would probably have that more like an essay style. ... But I would have a space instead of ... where it’d be like, why? I wouldn’t be like one to three, pick one. And then my ‘why’ would just be like an essay. And I would be like, explain your logic to me.” – Instructor 12*

Some instructors (*n* = 4) discussed wanting to modify the format of the 3DL question because they wish to assess their students on one concept at a time:

*“I would not use this question as written, because I think very few of my students would get it correct. I mean it’s a great question; it’s a multi-step; ... when I write my exam assessments, I’ve learned to only assess one thing at a time. So, I’m either going to assess them on the top part, like if it’s going to increase or decrease or not change, and then, my second question would assess them on what’s happening in the actual reaction. So, I wouldn’t try to assess them on two things at once.” – Instructor 7*

### Concept Inventory-Type Question

#### Fit with Assessment Context

Similarly, to the calculation-based question, most of the instructors (*n* = 13) would use the concept inventory-type question across all three assessment contexts (Figure 12). Sixteen of the 19 instructors placed this question in the exam/midterm portion of the Venn diagram, 14 in the homework portion, and 17 instructors in the in-class activity portion.

**Figure 12:**
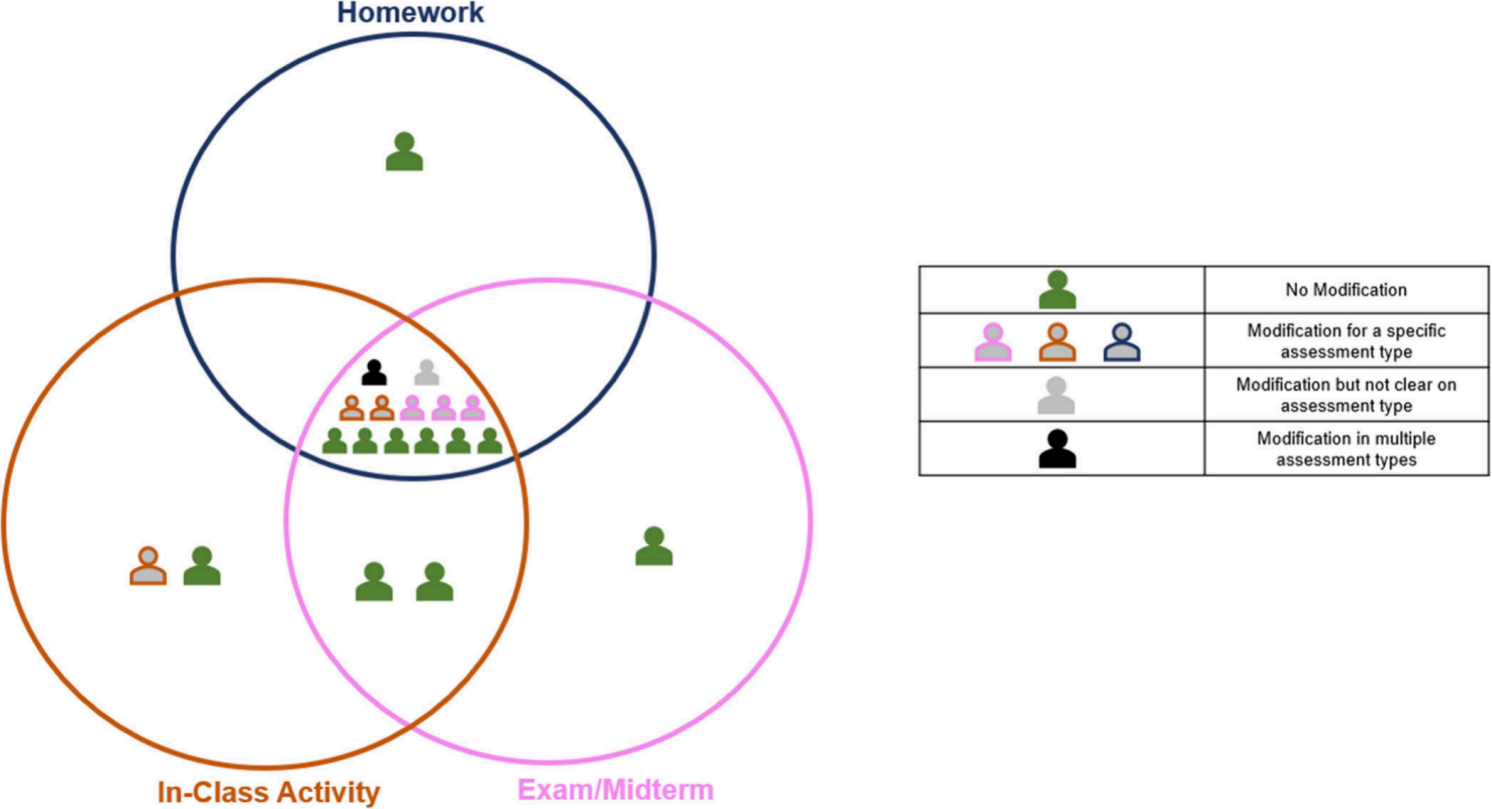
Instructors’ use for the concept inventory-type question.

Seven of the instructors indicated liking this question because of the representation and visualization of molecules:

*“And [concept inventory-type] ... I think I would use in any of the three. I like the pictorial questions, visual questions because that’s a great way for students to demonstrate their understanding.” – Instructor 10*

Another six instructors were drawn to the conceptual nature of the question:

*“It definitely is conceptual understanding ... I think I’m totally happy with this being a question in any three of those. The visual representation would lead me to introducing it early so that students can have a mental image of what they’re doing instead of just solely equations.” – Instructor 19*

Additionally, six instructors described this question being useful for an in-class activity, because “it could generate discussion in class” (Instructor 11).

It is also important to note that only two instructors explicitly stated that they would not use the concept inventory-type question for a particular assessment activity in their course, and their rationales for not using the question differed (see for their rationales).

#### Modifications

Almost half of the instructors (*n* = 8) would modify the concept inventory-type question, primarily for an in-class activity and exam/midterm (Figure 12). The modifications they described fell into three categories: modifying format (e.g., modifying the question type), modifying content (e.g., modifying the language used in the question), or unclear modifications (e.g., instructors mentioned wanting to modify the question, but the details of the modifications were unclear). Three instructors indicated wanting to modify the format of the question for an in-class activity (Figure 13). The most common change instructors described was to make the question more open-ended.

**Figure 13:**
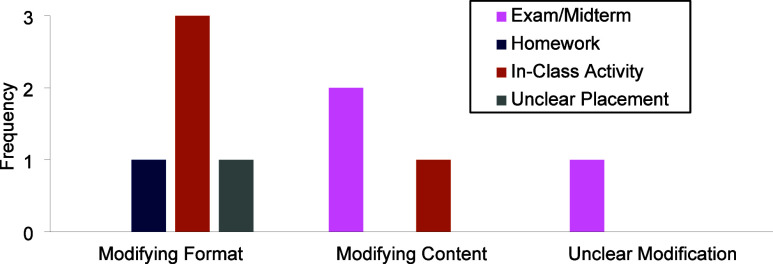
Instructors’ modification for the concept inventory-type question.

*“... if it were to be translated to an in-class activity it would be like a draw the picture of what it looks like ... not multiple choice but what do you think it looks like ...” – Instructor 2*

However, there was not a common rationale among the instructors for the need for this modification.

Additionally, three instructors described wanting to modify the content of the question with two instructors modifying the content for an exam/midterm and one instructor modifying the content for an in-class activity. These three instructors wanted to provide more clarity for what the spheres represent to minimize students’ confusion:

*“I would add, just for clarity*, *in addition to saying white spheres and black spheres, I would also put the image like in the legend ... That’s gonna be easier for students to read.” – Instructor 17*

## Discussion and Implications

The main goal of this study was to explore instructors’ perceptions of different types of questions and how they would utilize these questions within different assessment contexts in their course. This exploratory study provides an insight into chemistry instructors’ thinking regarding their assessment design.

### General Chemistry Instructors See a Fit of Research-Based Assessment Tools within Their Course’s Assessment plan

Most instructors indicated wanting to use the questions from research-based assessment tools (i.e., the concept inventory-type and 3DL questions) in different assessment contexts in their course. For the concept inventory-type question, instructors would use this question in any of the three assessment contexts in their course (e.g., exam, homework, and in-class activity). Instructors commonly appreciated the visualization and conceptual nature of this question. For the 3DL question, instructors varied more in how they would utilize this question in their course. While the majority would use it during class, around half of the instructors were reluctant to use this question in a homework and/or exam. Further indication that instructors saw a fit for these questions in their courses is that most of the modifications that were suggested for these questions did not compromise the intent of the question (e.g., no instructor argued against students thinking at the particulate level for the concept-inventory-type question).

Our findings thus indicate that most instructors would use these types of questions in at least one assessment context in their course. However, prior research has found that adoption of research-based assessment tools by chemistry instructors are minimal.^12,27^ The results of our study suggest that the lack of adoption of these tools may not be due to instructors’ dislike of these questions. A potential strategy to support instructors’ adoption of these assessment tools could be to emphasize their utility as assessment for learning (AfL) rather than solely as assessment of learning (AoL), which is often how their use is presented in the literature. Indeed, prior studies have found that instructors, like ours in this study, recognize the potential of these types of questions to support student learning.^41^ Moreover, prior studies have demonstrated that professional development programs can support the adoption of these tools by emphasizing their utility in supporting students.^42^

### General Chemistry Instructors Prefer Their Assessment Questions to Be Open-Ended

The most common modification that instructors wanted to make to all four questions was changing the multiple-choice format into an open-ended one. Instructors described wanting to make this change in format in order to see their students’ thinking and provide opportunities for students to show their work. Indeed, open-ended questions have been shown to be better suited to capture nuanced understandings of student thinking.^70^ Additionally, instructors saw value in some of these questions for stimulating student discussion and thought that switching to an open-ended format would enhance the potential for that discussion to take place. Of note, few instructors who taught large enrollment courses indicated wanting to switch to an open-format for the questions they were interested in using on exams. This is in alignment with prior literature,^59,60^ which demonstrates a heavy reliance on multiple-choice questions for exams in large enrollment courses.

Prior studies on STEM instructors’ adoption of 3DL questions have reported on the difficulties instructors have in writing multiple-choice 3DL questions.^41,42^ Similarly, concept-inventory-type questions require an instructor to have extensive knowledge of the various ways students can construe a concept in order to write appropriate distractors.^28^ Therefore, these two research-based assessment tools may be challenging for instructors to integrate into their large enrollment courses. The advent of artificial intelligence (AI) may provide, in the near-future, solutions to this problem. Indeed, AI could serve as a resource to support instructors in grading open-ended versions of these assessment questions and providing feedback to both students and instructors on the state of student learning.^71–73^ For example, a recent study demonstrated the capabilities of AI to analyze open-ended, written questions about the Lewis acid–base model.^74^

### General Chemistry Instructors Discussed Cognitive Level When Selecting Questions for Assessment Tasks

The most common rationale for instructors’ adoption or lack of adoption of the standard conceptual and 3DL questions was the cognitive demand of the question. This finding aligns with a study by Tomanek et al.,^75^ in which they explored factors influencing secondary science teachers’ evaluation and selection of formative assessment questions. The results of this study suggested that characteristics of the question, such as cognitive demand, influenced teachers’ utilization of different questions.

Prior research has indicated that exams and homework in science courses tend to use lower cognitive level assessment questions.^12,25,30–32^ We saw this in our study when some instructors preferred not to use the 3DL question on an exam due to the high cognitive demand of the question and their preference for assessing lower cognitive level content on exams. Interestingly, we also saw other instructors who praised the difficulty of the 3DL question. Our findings thus point to general chemistry instructors having different beliefs about the cognitive level that should be assessed in exams. In light of the findings from a prior study by Sanabria-Ríos and Bretz,^30^ which demonstrated that general chemistry instructors’ cognitive expectations for their students align with their assessment practices, future research should explore the origin and rationale behind general chemistry instructors’ cognitive expectations for their students and how this might affect instructor’s assessment design process. This work could lead to the development of professional development programs or policy recommendations that could help normalize the cognitive level expected and assessed in general chemistry courses and thus provide guidance on one aspect of assessment literacy that ought to be developed by general chemistry instructors, i.e., *knowledge of what to assess*.^45^

## Limitatons

During the interview process, the instructors were not provided with a description of the research-based assessment tools (3DL or concept-inventory type question) nor did this study capture if instructors understood the purpose for each type of question. If instructors were probed for the purpose behind the design of the research-based assessment tools, their preference for utilizing the question could change. Future research should explore instructors’ awareness of the purpose of these assessment tools, which could provide further insight into their slow uptake. Additionally, while the aim of the study was to recruit instructors from diverse demographics, there are limitations to the transferability of this work due to the small sample size. For example, this study could not explore whether context such as institution type impacted how instructors would use these assessment questions. Lastly, this study only explores a small aspect of chemistry instructors’ assessment literacy. Future studies are needed to fully understand the relationship between the various facets of assessment literacy and instructors’ choices to employ evidence-based assessment methods.

## Conclusion

This study explored general chemistry instructors’ preferences for the use of multiple-choice questions across various assessment contexts (homework, in-class activities, and exams/midterms), as well as their perspectives on modifying these questions. The findings reveal several key insights into instructors’ assessment design preferences and considerations. First, instructors demonstrated a general willingness to incorporate research-based assessment tools, such as concept inventory-type and 3DL questions, into their courses. These tools were valued for their ability to foster conceptual understanding, visualization, and cognitive engagement. However, instructors varied in their preferences for the specific assessment contexts in which these questions would be utilized. Notably, some instructors expressed reluctance to use high-cognitive-demand questions, such as 3DL questions, in exams due to lower cognitive expectations for their exams. Second, instructors frequently indicated a preference for modifying questions to an open-ended format to better assess students’ thinking and provide opportunities for students to show their work. While this aligns with the goal of promoting deeper learning, practical challenges—particularly in large enrollment courses—limit the feasibility of open-ended assessments. These challenges point to the potential for leveraging emerging technologies like AI to support the grading and feedback process for open-ended questions. Lastly, the study underscores the importance of cognitive demand in instructors’ decision-making about question use. While some instructors embraced higher-level questions to challenge students and stimulate discussion, others preferred lower-cognitive-demand questions, especially in time-constrained assessments like exams. This dichotomy highlights differing cognitive expectations of students, suggesting a need for professional development and policy guidance to help standardize assessment practices and expectations in general chemistry education. Overall, this study emphasizes the need for continued research on instructors’ assessment literacy to enhance the adoption of research-based assessment tools and promote higher-quality assessment in general chemistry courses.

## Supplementary Material





## Notes

The Supporting Information file contains the following: The Supporting Information is available at https://pubs.acs.org/doi/10.1021/acs.jchemed.5c00116.

Interview Protocol (S2) - Relevant portion of the protocol used to gather data from instructors, Assessment Analysis Framework (S3, S5) - Codebook for analyzing responses and summary of modifications made to the codebook, Standard Conceptual Questions (S7–S8) - Instructors’ rationales for using, not using, and modifying these assessment types, Calculation-based Questions (S9–S11) - Instructors’ rationales for using, not using, and modifying these assessment types, Three-Dimensional Learning (3DL) Questions (S11–S12) - Instructors’ rationales for using, not using, and modifying these assessment types, Concept Inventory-Type Questions (S13–S14) - Instructors’ rationales for using, not using, and modifying these assessment types (PDF, DOCX)
