# Supplementary material for: Unlocking Instructors’ Assessment Insights: General Chemistry Instructors’ Perspectives on Types of Questions and their Classroom Application
Source: J Chem Educ. 2025 Sep 3;102(10):4200–13. doi: 10.1021/acs.jchemed.5c00116 (PMC12529954; doi:10.1021/acs.jchemed.5c00116)
Supplement: Supplementary file 2 [file ed5c00116_si_002.docx]

**Supplemental Information**

**For**

**Unlocking Instructors’ Assessment Insights: General Chemistry Instructors’ Perspectives on Types of Assessment Questions and their Classroom Application**

Emily A. Kable^1^, Ying Wang^1^, Lu Shi^2^, Marilyne Stains^1^*

^1^ Department of Chemistry, University of Virginia, Charlottesville, Virginia, 22904, United States

^2^ STEM Education Innovation and Research Institute, Indiana University Indianapolis, Indianapolis, Indiana 27412, United States

*Corresponding author’s email: [mstains@virginia.edu](mailto:mstains@virginia.edu)

Contents

[A. Relevant Portion of the Interview Protocol 2](#_Toc175642941)

[B. Assessment Codebook 3](#_Toc175642942)

[C. Summary of modifications made to assessment codebook 5](#_Toc175642943)

[D. Instructors’ rationale for using Standard Conceptual question 7](#_Toc175642944)

[E. Instructors’ rationale for not using Standard Conceptual question 8](#_Toc175642945)

[F. Instructors’ modification for Standard Conceptual question 8](#_Toc175642946)

[G. Instructors' rationale for using Calculation-based question 9](#_Toc175642947)

[H. Instructors’ rationale for not using Calculation-based question 10](#_Toc175642948)

[I. Instructors’ modifications for Calculation-based question 11](#_Toc175642949)

[J. Instructors' rationale for using 3DL question 11](#_Toc175642950)

[K. Instructors' rationale for not using 3DL question 12](#_Toc175642951)

[L. Instructors’ modifications for 3DL question 12](#_Toc175642952)

[M. Instructors' rationale for using Concept Inventory-Type question 13](#_Toc175642953)

[N. Instructors’ rationale for not using Concept Inventory-Type question 14](#_Toc175642954)

[O. Instructors’ modifications for Concept Inventory-Type question 14](#_Toc175642955)

# A. Relevant Portion of the Interview Protocol

In the last part of the interview, we are interested in understanding the type of questions that appeal to faculty and why they appeal to them.

We provide here a set of questions that we found in textbooks, databases, and other resources that would be available to an instructor. All aim at assessing students’ understanding of Le Chatelier’s principle.

We would like you to consider each question and think about whether you would use it to assess your students and if so for what type of assessment: in-class, homework, mid-terms/final exams). We will use this Venn diagram to help identify the context in which you would use the questions that you are interested in. Just use the number associated with the question and place it on the Venn diagram.

As you consider each question, please think aloud your thought processes. Not all questions have to be placed in the diagram. Only those that you like.

(If you put the question in the line part which means the questions fit all three assessment scenarios; the dot sign part means the questions fit both homework and exam/quiz; the star sign part means the questions fit both homework and in-class activity; the cross sign part means the questions fit both in-class activity and exam/quiz.)

1. Why do you like this question?
2. Why did you put this question under XX scenarios? (Reasoning).
3. Would you modify it before using it? Why? How?
4.
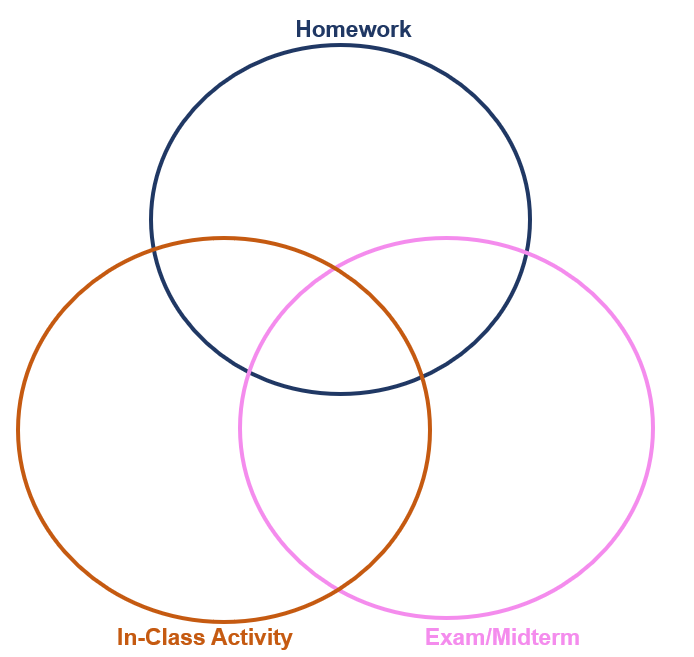
Here are the questions that you didn’t choose. Why did you not choose them?

#

# B. Assessment Codebook

|  | **Code** | **Definition** |
| --- | --- | --- |
| Question Number: every code needs to be associated with a question number | Q1 | every code needs to be associated with a question number from interview protocol |
|  | Q2 | every code needs to be associated with a question number from interview protocol |
|  | Q3 | every code needs to be associated with a question number from interview protocol |
|  | Q4 | every code needs to be associated with a question number from interview protocol |
| Venn Diagram: every code needs to be associated with assessment type | Homework | The instructor explicitly mentions this question with homework. |
|  | Midterm/Exam | The instructor explicitly mentions this question with a midterm or exam. |
|  | In-Class Activity | The instructor explicitly mentions this question with “in-class” and/or in a group setting. |
|  | Quiz | The instructor mentions this question with a quiz. Except for mentioning the question as a team quiz, that will be coded as an in-class activity. |
|  | Unclear Placement | The instructor mentions the question but does not explicitly state where they would use or not use the question. |
| Assessment Selection | Use and like with modification | The instructor would use and like the question but would want to modify the question. |
|  | Use and like without modification | The instructor would use and like the question and use as is. |
|  | Not use and do not like | The instructor would not use and does not like the question. |
|  | Use if they had to with modification | The instructor mentioned they would use the question the question if they had to, but they would want to modify the question. (if the instructor shows any dislike for the question, but then mentions how they would use it if modified) |
|  | Use if they had to without modification | The instructor mentioned they would use the question if they had to and use as is. (If the instructor shows dislike for the question, but then mentions how they would use it-it would be coded here.) |
| Rationale for Selection | Applicable question | The instructor prefers questions that allows students to learn the skills needed to calculate something they might need in a lab. Alternatively, the instructor states that they prefer questions that show that students can apply concepts to a chemical system. |
|  | Conceptual question | The instructor explicitly mentions “conceptual” (conceptual understanding or conceptual question for example) |
|  | Discuss | The instructor believes the question would allow for students to work together and discuss to come to an understanding of the topic. The instructor could also mention wanting their students to make predictions about the answers and then discussing. The instructor might also want to be present or a teaching assistant present to help their students work through the question. |
| Rationale for Selection cont. | Easy grading | The instructor believes the question will be easy to grade, or they like the structure of the multiple-choice format, and/or could grade for accuracy rather than attempt. |
|  | Easy to answer | The instructor thinks the question is fundamental, basic, straightforward, or concept check type of question. The instructor might also think the question is easy for students to guess the answer, the question might not take a lot of time to complete, and/or feels that once students have seen the question, it is too straightforward to use it again. |
|  | Immediate feedback | The question provides a way to give students immediate feedback. |
|  | Individual work | The instructor believes that the students can work through the question on their own. |
|  | Longer question | The question will take students more time to work through it, or the question itself is lengthier (more words). Also, if the instructor mentions that there could be a time constraint and/or wanting their students to have more time to think about it. |
| Rationale for Selection cont. | Multi-level question | This question takes more skill to solve, higher-order thinking, asks students to explain, or multiple concepts being assessed. The instructor might also think the question has too many steps for students to complete or confusing language. The instructor could also mention the broken-down structure of the question itself. |
|  | Question provided appropriate information | This question provided appropriate content components in the question such as the instructor likes the concepts being assessed or the information provided in the question. If an instructor talks about the picture diagrams, this is not coded here. |
|  | Representation | The question is a visual question and has diagrams for students to use. The instructor could also not like the drawing in the question or the representation of the molecules. |
|  | Seen or used previously | The instructor or students have seen and/or used the question previously. The instructor might also want their students to have seen the question previously to use it in the situation they are discussing. |
|  | Student thinking | The question can help provide insight into if they got the question wrong, or tell them they are on the right track. Alternatively, the instructor wants students to explain to see their thinking or perform a calculation through a problem. |
|  | Too much room for small errors | The question has too many opportunities for students to make small errors. |
| Ways of Modification | Modifying Content | The instructor wants to make modifications to the language being used in the question and/or the answer choices. (**NOTE**: this code included the sub codes of “Modification to the language being used in the question” and “Modification to the language being used in the answer choices”.) |
|  | Modifying Format | The instructor wants to make a modification to the question type, number of answer choices, break-up the question, or combine the question. (**NOTE**: this code included the sub codes of “modification to question type”, “modification to add the number of answer choices”, “modification to reduce the number of answer choices”, “Modification to scaffold the question”, “Modification to break-up the question”, and “Modification to combine the question”) |
|  | None Modification | The instructor does not want to modify the question. |
|  | Unclear Modification | The instructor wants to modify the question, but they did not specify how they want to modify the question. |

# C. Summary of modifications made to assessment codebook

| **Changes made after Version 1** | - Added “Unclear Placement” code to the Venn diagram category. - “Use/Like with modification” was changed to “Use and like with modification” for clarity. - EAK and YW decided not to include a “modification rationale” as a code, but instead as a label to see if enough instructors discussed this. - Combined “Question provides insight” and “Understanding the question” to call it “Metacognition” due to similarities in codes. - Combined “No opportunity to show work” with “Only tests a concept without explanation or calculation” due to similarities in codes. - Deleted “Good content question” as it was capturing the same thing as “Applicable question” - Combined “Too many unit conversions”, “Question is a lot of work’, and “Question is too long” to form “Question takes too long”. The question was then clarified the definition to include the question length being long, or a time constraint. - Clarified the definition for “Question is too complication” by providing examples. - Clarified the title and definition for “Instructor bad experience” to indicate that it was a bad experience as student. - Clarified the definition for “Easy guessing” to indicate that instructors think it is easy due to being multiple choice. |
| --- | --- |
| **Changes made after Version 2** | - EAK and YW decided to shorten codebook by not distinguishing rationale specific for instructors’ use or nonuse. - Clarified definition for “Question provided appropriate information” to not include diagrams as that is already being captured separately. - Combined “Time to think about question”, “Longer question”, and “Question too long” into “Longer question” due to similarities in definition and clarified the definition. |
| **Changes made after Version 3** | - Clarified the definition for “Question provided appropriate information” to provide examples. - Clarified the definitions for “Discussion question” and “Student thinking” to distinguish between the two codes, and “Student thinking” was changed to “Opportunities for student thinking”. - “Good prediction question” was combined into “Discussion question” due similarities in definitions. - “Applicable question” definition was edited for clarity. |
| **Changes made after Version 4** | - Clarified the definitions for “Use if they had to with modification” and “Use if they had to without modification”. - Clarified the definition for “Seen/Used previously” by including examples. - Clarified the definition for “Longer question” to alter language. - Made “Modification to adjust the information provided in the question based on scenarios of implementation” a subcode of “Modification to the language being used in the question” due to similarities in codes. (**NOTE:** this subcode was used as a label) |
| **Changes made after Version 5** | - Combined “Question is too complicated” with “Multi level question” due to similarities in definition. - Combined “Only tests a concept without explanation or calculation” with “Good question for concept check” due to similarities in definitions. - Clarified the definition for “Longer question” to alter language. |
| **Changes made after Version 6** | - Altered the name for “Good question for concept check” to “Concept check”. |
| **Changes made after Version 7** | - Combined “Broken down question” into “Multi-level question” and clarified the definition for clarity. - Combined “Calculation-based question” into “Opportunities for student thinking” and clarified definition for clarity. - Combined “Too much drawing” into “Visual question” due to similarities in definition. - Clarified the definition for “Concept check” to distinguish this code from “Conceptual question”. |
| **EAK, YW, and MS met to group codes into potential categories** | - “Easy to grade” and “Multiple choice question” were combined into “Easy grading”. - “Metacognition” and “Opportunities for student thinking” were combined into “Student thinking”. - “Representation of molecules” and “Visual question” were combined into “Representation”. - “Easy guessing”, “Too straight forward if student seen before”, “Quick question”, “Concept check” were combined into “Easy to answer”. - “Instructor help with question” and “Discussion question” were combined into “Discuss”. |

# D. Instructors’ rationale for using Standard Conceptual question

**Note:** Each cell in the table represents the number of instructors who gave a specific rationale for using the standard conceptual question in an assessment type. Since instructors can share the same rationale across different assessment types, the rows are not mutually exclusive. The ‘Total Participants’ column indicates the number of individual instructors who expressed each rationale across the assessment types. The ‘Total Participants’ column excludes the ‘Quiz’ category as that was not analyzed further in this study.

| **Rationale** | **Quiz** | **Homework** | **Exam/Midterm** | **In-Class Activity** | **Total Participants** |
| --- | --- | --- | --- | --- | --- |
| Immediate Feedback | 0 | 0 | 0 | 0 | 0 |
| Individual work | 0 | 0 | 0 | 0 | 0 |
| Too much room for small errors | 0 | 0 | 0 | 0 | 0 |
| Applicable question | 0 | 0 | 0 | 0 | 0 |
| Seen or used previously | 0 | 0 | 0 | 0 | 0 |
| Question provided appropriate information | 0 | 0 | 2 | 0 | 2 |
| Easy grading | 0 | 3 | 3 | 1 | 5 |
| Conceptual question | 0 | 0 | 1 | 0 | 1 |
| Longer question | 0 | 0 | 0 | 0 | 0 |
| Student thinking | 0 | 1 | 1 | 0 | 1 |
| Easy to answer | 2 | 3 | 7 | 5 | 11 |
| Representation | 0 | 0 | 0 | 0 | 0 |
| Discuss | 0 | 0 | 0 | 1 | 1 |
| Multi-level question | 0 | 0 | 0 | 0 | 0 |

# E. Instructors’ rationale for not using Standard Conceptual question

**Note:** Each cell in the table represents the number of instructors who gave a specific rationale for not using the standard conceptual question in an assessment type. Since instructors can share the same rationale across different assessment types, the rows are not mutually exclusive. The ‘Total Participants’ column indicates the number of individual instructors who expressed each rationale across the assessment types. The ‘Total Participants’ column excludes the ‘Quiz’ category as that was not analyzed further in this study.

| **Rationale** | **Quiz** | **Homework** | **Exam/Midterm** | **In-Class Activity** | **Total Participants** |
| --- | --- | --- | --- | --- | --- |
| Immediate Feedback | 0 | 0 | 0 | 0 | 0 |
| Individual work | 0 | 0 | 0 | 0 | 0 |
| Too much room for small errors | 0 | 0 | 0 | 0 | 0 |
| Applicable question | 0 | 0 | 0 | 0 | 0 |
| Seen or used previously | 0 | 0 | 0 | 0 | 0 |
| Question provided appropriate information | 0 | 0 | 0 | 0 | 0 |
| Easy grading | 0 | 0 | 0 | 1 | 1 |
| Conceptual question | 0 | 0 | 0 | 0 | 0 |
| Longer question | 0 | 0 | 0 | 0 | 0 |
| Student thinking | 0 | 0 | 0 | 0 | 0 |
| Easy to answer | 0 | 0 | 2 | 1 | 3 |
| Representation | 0 | 0 | 0 | 0 | 0 |
| Discuss | 0 | 0 | 0 | 0 | 0 |
| Multi-level question | 0 | 0 | 0 | 0 | 0 |

# F. Instructors’ modification for Standard Conceptual question

**Note:** Each cell in the table represents the number of instructors who described their desired modification type for using the standard conceptual question for a specific assessment type. Since instructors can share the same modification across different assessment types, the rows are not mutually exclusive. The ‘Total Participants’ column indicates the number of individual instructors who expressed each modification across the assessment types.

| **Types of Modification** | **Homework** | **Exam/Midterm** | **In-Class Activity** | **Unclear Placement** | **Total Participants** |
| --- | --- | --- | --- | --- | --- |
| Modifying Format | 2 | 2 | 2 | 0 | 4 |
| Modifying Content | 1 | 2 | 0 | 1 | 4 |
| Unclear Modification | 0 | 0 | 0 | 0 | 0 |

# G. Instructors' rationale for using Calculation-based question

| **Rationale** | **Quiz** | **Homework** | **Exam/Midterm** | **In-Class Activity** | **Total Participants** |
| --- | --- | --- | --- | --- | --- |
| Immediate Feedback | 0 | 0 | 0 | 0 | 0 |
| Individual work | 0 | 0 | 0 | 0 | 0 |
| Too much room for small errors | 0 | 0 | 0 | 0 | 0 |
| Applicable question | 0 | 0 | 1 | 0 | 1 |
| Seen or used previously | 0 | 0 | 1 | 0 | 1 |
| Question provided appropriate information | 0 | 1 | 1 | 1 | 1 |
| Easy grading | 0 | 0 | 1 | 0 | 1 |
| Conceptual question | 0 | 0 | 0 | 0 | 0 |
| Longer question | 0 | 4 | 0 | 2 | 4 |
| Student thinking | 0 | 1 | 2 | 4 | 5 |
| Easy to answer | 0 | 1 | 1 | 1 | 1 |
| Representation | 0 | 0 | 0 | 0 | 0 |
| Discuss | 0 | 2 | 0 | 5 | 6 |
| Multi-level question | 0 | 2 | 2 | 2 | 3 |

**Note:** Each cell in the table represents the number of instructors who gave a specific rationale for using the calculation-based question in an assessment type. Since instructors can share the same rationale across different assessment types, the rows are not mutually exclusive. The ‘Total Participants’ column indicates the number of individual instructors who expressed each rationale across the assessment types. The ‘Total Participants’ column excludes the ‘Quiz’ category as that was not analyzed further in this study.

# H. Instructors’ rationale for not using Calculation-based question

**Note:** Each cell in the table represents the number of instructors who gave a specific rationale for not using the calculation in an assessment type. Since instructors can share the same rationale across different assessment types, the rows are not mutually exclusive. The ‘Total Participants’ column indicates the number of individual instructors who expressed each rationale across the assessment types. The ‘Total Participants’ column excludes the ‘Quiz’ category as that was not analyzed further in this study.

| **Rationale** | **Quiz** | **Homework** | **Exam/Midterm** | **In-Class Activity** | **Total Participants** |
| --- | --- | --- | --- | --- | --- |
| Immediate feedback | 0 | 0 | 0 | 0 | 0 |
| Individual work | 0 | 0 | 0 | 0 | 0 |
| Too much room for small errors | 0 | 0 | 1 | 0 | 1 |
| Applicable question | 0 | 0 | 0 | 0 | 0 |
| Seen or used previously | 0 | 0 | 0 | 0 | 0 |
| Question provided appropriate information | 0 | 0 | 0 | 0 | 0 |
| Easy grading | 0 | 0 | 0 | 0 | 0 |
| Conceptual question | 0 | 0 | 0 | 0 | 0 |
| Longer question | 1 | 0 | 1 | 2 | 2 |
| Student thinking | 0 | 0 | 1 | 0 | 1 |
| Easy to answer | 0 | 0 | 0 | 0 | 0 |
| Representation | 0 | 0 | 0 | 0 | 0 |
| Discuss | 0 | 0 | 0 | 0 | 0 |
| Multi-level question | 1 | 0 | 1 | 0 | 1 |

# I. Instructors’ modifications for Calculation-based question

**Note:** Each cell in the table represents the number of instructors who described their desired modification type for using the calculation-based question for a specific assessment type. Since instructors can share the same modification across different assessment types, the rows are not mutually exclusive. The ‘Total Participants’ column indicates the number of individual instructors who expressed each modification across the assessment types.

| **Types of Modifications** | **Homework** | **Exam/Midterm** | **In-Class Activity** | **Unclear Placement** | **Total Participants** |
| --- | --- | --- | --- | --- | --- |
| Modifying Format | 4 | 6 | 7 | 1 | 12 |
| Modifying Content | 1 | 1 | 1 | 2 | 4 |
| Unclear Modification | 0 | 1 | 0 | 0 | 1 |

# J. Instructors' rationale for using 3DL question

**Note:** Each cell in the table represents the number of instructors who gave a specific rationale for using the 3DL question in an assessment type. Since instructors can share the same rationale across different assessment types, the rows are not mutually exclusive. The ‘Total Participants’ column indicates the number of individual instructors who expressed each rationale across the assessment types. The ‘Total Participants’ column excludes the ‘Quiz’ category as that was not analyzed further in this study.

| **Rationale** | **Quiz** | **Homework** | **Exam/Midterm** | **In-Class Activity** | **Total Participants** |
| --- | --- | --- | --- | --- | --- |
| Immediate Feedback | 0 | 0 | 0 | 0 | 0 |
| Individual Work | 0 | 0 | 0 | 0 | 0 |
| Too much room for small errors | 0 | 0 | 0 | 0 | 0 |
| Applicable question | 0 | 0 | 0 | 0 | 0 |
| Seen or used previously | 0 | 0 | 0 | 0 | 0 |
| Question provided appropriate information | 0 | 0 | 0 | 0 | 0 |
| Easy grading | 0 | 0 | 1 | 0 | 1 |
| Conceptual question | 0 | 0 | 1 | 1 | 2 |
| Longer question | 0 | 0 | 1 | 0 | 1 |
| Student thinking | 0 | 1 | 1 | 1 | 1 |
| Easy to answer | 0 | 0 | 0 | 1 | 1 |
| Representation | 0 | 0 | 0 | 0 | 0 |
| Discuss | 0 | 0 | 0 | 6 | 6 |
| Multi-level question | 0 | 2 | 6 | 5 | 11 |

# K. Instructors' rationale for not using 3DL question

**Note:** Each cell in the table represents the number of instructors who gave a specific rationale for not using the 3DL question in an assessment type. Since instructors can share the same rationale across different assessment types, the rows are not mutually exclusive. The ‘Total Participants’ column indicates the number of individual instructors who expressed each rationale across the assessment types. The ‘Total Participants’ column excludes the ‘Quiz’ category as that was not analyzed further in this study.

| **Rationale** | **Quiz** | **Homework** | **Exam/Midterm** | **In-Class Activity** | **Total Participants** |
| --- | --- | --- | --- | --- | --- |
| Immediate Feedback | 0 | 0 | 0 | 0 | 0 |
| Individual Work | 0 | 0 | 0 | 0 | 0 |
| Too much room for small errors | 0 | 0 | 0 | 0 | 0 |
| Applicable question | 0 | 0 | 0 | 0 | 0 |
| Seen or used previously | 0 | 0 | 1 | 0 | 1 |
| Question provided appropriate information | 0 | 0 | 0 | 0 | 0 |
| Easy grading | 0 | 0 | 0 | 0 | 0 |
| Conceptual question | 0 | 0 | 0 | 0 | 0 |
| Longer question | 1 | 0 | 0 | 1 | 1 |
| Student thinking | 0 | 0 | 0 | 0 | 0 |
| Easy to answer | 0 | 0 | 0 | 0 | 0 |
| Representation | 0 | 0 | 0 | 0 | 0 |
| Discuss | 0 | 0 | 0 | 0 | 0 |
| Multi-level question | 1 | 2 | 4 | 1 | 6 |

# L. Instructors’ modifications for 3DL question

**Note:** Each cell in the table represents the number of instructors who described their desired modification type for using the 3DL question for a specific assessment type. Since instructors can share the same modification across different assessment types, the rows are not mutually exclusive. The ‘Total Participants’ column indicates the number of individual instructors who expressed each modification across the assessment types.

| **Types of Modification** | **Homework** | **Exam/Midterm** | **In-Class Activity** | **Unclear Placement** | **Total Participants** |
| --- | --- | --- | --- | --- | --- |
| Modifying Format | 2 | 7 | 1 | 0 | 7 |
| Modifying Content | 0 | 2 | 1 | 0 | 3 |
| Unclear Modification | 0 | 1 | 0 | 0 | 1 |

# M. Instructors' rationale for using Concept Inventory-Type question

**Note:** Each cell in the table represents the number of instructors who gave a specific rationale for using the concept inventory-type question in an assessment type. Since instructors can share the same rationale across different assessment types, the rows are not mutually exclusive. The ‘Total Participants’ column indicates the number of individual instructors who expressed each rationale across the assessment types. The ‘Total Participants’ column excludes the ‘Quiz’ category as that was not analyzed further in this study.

| **Rationale** | **Quiz** | **Homework** | **Exam/Midterm** | **In-Class Activity** | **Total Participants** |
| --- | --- | --- | --- | --- | --- |
| Immediate Feedback | 0 | 0 | 0 | 1 | 1 |
| Individual Work | 0 | 1 | 1 | 0 | 1 |
| Too much room for small errors | 0 | 0 | 0 | 0 | 0 |
| Applicable question | 0 | 0 | 0 | 0 | 0 |
| Seen or used previously | 0 | 0 | 2 | 0 | 2 |
| Question provided appropriate information | 0 | 1 | 0 | 1 | 1 |
| Easy grading | 0 | 1 | 3 | 0 | 3 |
| Conceptual question | 0 | 4 | 3 | 5 | 6 |
| Longer question | 0 | 2 | 1 | 1 | 2 |
| Student thinking | 0 | 2 | 1 | 2 | 3 |
| Easy to answer | 0 | 0 | 1 | 0 | 1 |
| Representation | 0 | 5 | 5 | 7 | 7 |
| Discuss | 0 | 1 | 0 | 6 | 6 |
| Multi-level question | 0 | 1 | 1 | 1 | 2 |

# N. Instructors’ rationale for not using Concept Inventory-Type question

**Note:** Each cell in the table represents the number of instructors who gave a specific rationale for not using the concept inventory-type question in an assessment type. Since instructors can share the same rationale across different assessment types, the rows are not mutually exclusive. The ‘Total Participants’ column indicates the number of individual instructors who expressed each rationale across the assessment types. The ‘Total Participants’ column excludes the ‘Quiz’ category as that was not analyzed further in this study.

| **Rationale** | **Quiz** | **Homework** | **Exam/Midterm** | **In-Class Activity** | **Total Participants** |
| --- | --- | --- | --- | --- | --- |
| Immediate Feedback | 0 | 0 | 0 | 0 | 0 |
| Individual Work | 0 | 0 | 0 | 0 | 0 |
| Too much room for small errors | 0 | 0 | 0 | 0 | 0 |
| Applicable question | 0 | 0 | 0 | 0 | 0 |
| Seen or used previously | 0 | 0 | 0 | 0 | 0 |
| Question provided appropriate information | 0 | 0 | 0 | 0 | 0 |
| Easy grading | 0 | 0 | 0 | 0 | 0 |
| Conceptual question | 0 | 0 | 0 | 0 | 0 |
| Longer question | 0 | 0 | 1 | 0 | 1 |
| Student thinking | 0 | 1 | 1 | 0 | 1 |
| Easy to answer | 0 | 0 | 1 | 0 | 1 |
| Representation | 0 | 0 | 1 | 1 | 1 |
| Discuss | 0 | 0 | 0 | 0 | 0 |
| Multi-level question | 0 | 0 | 0 | 0 | 0 |

# O. Instructors’ modifications for Concept Inventory-Type question

**Note:** Each cell in the table represents the number of instructors who described their desired modification type for using the concept inventory-type question for a specific assessment type. Since instructors can share the same modification across different assessment types, the rows are not mutually exclusive. The ‘Total Participants’ column indicates the number of individual instructors who expressed each modification across the assessment types.

| **Types of Modification** | **Homework** | **Exam/Midterm** | **In-Class Activity** | **Unclear Placement** | **Total Participants** |
| --- | --- | --- | --- | --- | --- |
| Modifying Format | 1 | 0 | 3 | 1 | 4 |
| Modifying Content | 0 | 2 | 1 | 0 | 3 |
| Unclear Modification | 0 | 1 | 0 | 0 | 1 |
